# Recovery of Cognitive Dysfunction via Orally Administered Redox-Polymer Nanotherapeutics in SAMP8 Mice

**DOI:** 10.1371/journal.pone.0126013

**Published:** 2015-05-08

**Authors:** Pennapa Chonpathompikunlert, Toru Yoshitomi, Long Binh Vong, Natsuka Imaizumi, Yuki Ozaki, Yukio Nagasaki

**Affiliations:** 1 Department of Materials Sciences, Graduate School of Pure and Applied Sciences, University of Tsukuba, Tennoudai 1-1-1, Tsukuba, Ibaraki 305–8573, Japan; 2 Master’s School of Medical Sciences, Graduate School of Comprehensive Human Sciences, University of Tsukuba, Tennoudai 1-1-1, Tsukuba, Ibaraki 305–8573, Japan; 3 Satellite Laboratory, International Center for Materials Nanoarchitechtonics (WPI-MANA), National Institute for Materials Sciences (NIMS), University of Tsukuba, Tennoudai 1-1-1, Tsukuba, Ibaraki 305–8573, Japan; Institute for Frontier Medical Sciences, Kyoto University, JAPAN

## Abstract

Excessively generated reactive oxygen species are associated with age-related neurodegenerative diseases. We investigated whether scavenging of reactive oxygen species in the brain by orally administered redox nanoparticles, prepared by self-assembly of redox polymers possessing antioxidant nitroxide radicals, facilitates the recovery of cognition in 17-week-old senescence-accelerated prone (SAMP8) mice. The redox polymer was delivered to the brain after oral administration of redox nanoparticles via a disintegration of the nanoparticles in the stomach and absorption of the redox polymer at small intestine to the blood. After treatment for one month, levels of oxidative stress in the brain of SAMP8 mice were remarkably reduced by treatment with redox nanoparticles, compared to that observed with low-molecular-weight nitroxide radicals, resulting in the amelioration of cognitive impairment with increased numbers of surviving neurons. Additionally, treatment by redox nanoparticles did not show any detectable toxicity. These findings indicate the potential of redox polymer nanotherapeutics for treatment of the neurodegenerative diseases.

## Introduction

Aging increases the risk of neurodegenerative diseases, such as Alzheimer’s disease (AD), which mostly affect quality of life in the elderly. Although the average human life span has increased because of progress in medical and health care, the socioeconomic burden of the elderly is a concern in developed countries. Oxidative stress caused by overproduction of reactive oxygen species (ROS) is well known as one of the direct causes of aging. Under normal physiological conditions, ROS can be scavenged by endogenous antioxidant-defense systems including superoxide dismutase (SOD), catalase (CAT), and glutathione peroxidase (GPx). With advancing age, however, the production of ROS dramatically increases, and endogenous antioxidants fail to completely scavenge all of the ROS, followed by production of oxidative components. An increase in the oxidative stress in the brain is reported to be involved in aging-related neural dysfunction and/or learning and memory deficiency [1]. Previous studies have suggested that an increase in the expression of pro-inflammatory cytokines, such as interleukin (IL)-1β, IL-6, and tumor necrosis factor-α (TNF-α), in the brain is involved in aging-related neural dysfunction and/or learning and memory deficiency in animals. Although the promising low-molecular-weight (LMW) antioxidant, vitamin E, was reported to show slight efficacy such as slowing of functional decline, in clinical trials of AD, a complete recovery was not observed [2, 3].

Recently, drug delivery systems using nanomedicines have attracted much attention in the areas of diagnosis and therapy, and they are expected to have various clinical applications [4]. For example, since nanoparticles with long-term blood circulation specifically accumulate in tumor tissues after intravenous administration, referred to as the enhanced permeability and retention (EPR) effect [5], controlled drug release from nanoparticles at tumor sites increases the therapeutic effects of the anticancer drug and suppresses its severe adverse effects [6]. However, for the treatment of chronic diseases, such as AD, oral medications are preferred owing to the convenient and non-invasive method for patients. Most nanomedicines are not used as orally administered drugs for systemic diseases because nanoparticles between the sizes of 10 and 100 nm are not absorbed via the gastrointestinal tract [7]. If an orally administered drug with nanomedicine-like characteristics is absorbed in the blood, it may be an ideal oral medication for chronic diseases. Recently, we have proposed “redox polymer nanotherapeutics” using amphiphilic block copolymer, poly(ethylene glycol)-*b*-poly[4-(2,2,6,6-tetramethylpiperidine-1-oxyl)aminomethylstyrene] (MeO-PEG-*b*-PMNT) (10 kDa, referred to as a redox polymer) [8–10]. This redox polymer possesses nitroxide radicals, as ROS-scavengers, in the hydrophobic segments via covalent linkages and forms a polymeric micelle under physiological conditions, which confines the nitroxide radicals in its core and is 40 nm in diameter (referred to as the redox nanoparticle [RNP^N^]). We have confirmed that RNP^N^ disintegrates after intravenous administration in response to low pH environments, such as ischemic, inflamed, and tumor tissues, owing to protonation of amino groups of the hydrophobic segment, resulting in increased ROS scavenging activity because of an exposure of the nitroxide radicals from the RNP^N^ core [11]. Thus far, we have confirmed the remarkable therapeutic effect of intravenously administered RNP^N^ for various oxidative stress injuries such as ischemia-reperfusion injuries of the kidney [11], brain [12], and heart (Asanuma, et al., submitted for publication), and intracerebral hemorrhage [13]. Although LMW nitroxide radicals cause severe adverse effects because of internalization in healthy cells and disturbance of the important electron transport chain in mitochondria [14], redox polymer nanoparticles do not cause adverse effects because of no internalization in healthy cells such as blood cells [15] and colon mucosal cells [16]. Since we have already confirmed that RNP^N^ shows an anti-apoptotic effect on amyloid beta-induced cell death *in vitro* [17], in this study, we applied RNP^N^ as an orally administered redox polymer nanotherapeutics for the treatment of senescence-accelerated neural dysfunction. Owing to the low viscosity of the nanoparticle solution, this solution can be received easily. Considering the long-term treatment of senescence-accelerated neurodegenerative diseases such as AD, this advantage helps patients to take medicine. After oral administration, it disintegrated under acidic conditions in the stomach, followed by absorption of the redox polymers into the bloodstream across the intestinal epithelium. Because of the covalent linkages of nitroxide radicals to redox polymers, nitroxide radicals are absorbed together with the polymer in the bloodstream. In this study, we examined the blood uptake of the redox polymer and its delivery in the brain after oral administration of RNP^N^ in wild-type ICR mice. Furthermore, we evaluated whether our redox polymer nanotherapeutics was effective in senescence-accelerated prone (SAMP8) mice, which are suitable models of the accelerated senescence with early learning and memory deficits [18, 19].

## Methods

### Preparation of the RNP^N^

The RNP^N^ was prepared by self-assembly of redox polymers (MW [PEG] = 5,500 Da; MW [PMNT] = 4,500 Da) by using the dialysis method reported previously [20]. Please see the supporting information for a comprehensive description of the methods.

### Animals

Male ICR, male SAMP8 and SAMR1 mice were used in this study. They were housed in the experimental animal center of University of Tsukuba under controlled temperature (23 ± 1°C), humidity (50 ± 5%) and lighting (12 h light/dark cycles). The animals had free access to food and water. All the experiments were carried out in accordance with the guidelines for animal care and use of Japan and were approved by the animal ethics committee of the Institutional Animal Experiment Committee of the University of Tsukuba and in accordance with the Regulation for Animal Experiments in our university and the Fundamental Guideline for Proper Conduct of Animal Experiments and Related Activities in Academic Research Institutions under the jurisdiction of the Ministry of Education, Culture, Sports, Science, and Technology.

### Evaluation of the delivery of redox polymer to the brain of wild-type ICR mice after oral administration of RNP^N^

Six-week-old male ICR mice (Charles River Japan, Inc., Kanagawa, Japan) were used for the evaluation of the delivery of the redox polymer to the brain. Please see detailed methods in the supporting information.

### Evaluation of therapeutic effects of redox polymer nanotherapeutics

Seventeen-week-old male SAMP8 (body weight; 30 ± 2.0 g) and 17-week-old male SAMR1 mice (body weight; 35 ± 2.0 g) were purchased from Japan SLC, Inc. (Shizuoka, Japan) for this study. All of the SAMR1 and SAMP8 mice were trained using the Morris water maze for seven days and using an open field instrument for three days before the mice were randomly divided into five groups (10 mice/group). Two hundred microliters of RNP^N^ (60 mg/mL) were orally administered to SAMP8 mice at a dose of 300 mg/(kg·d) (nitroxide radical concentration: 42.5 mg/(kg·d)) for four consecutive weeks. Blank micelles (nanoparticles without nitroxide radicals in the core: 60 mg/mL) and 4-hydroxy-2,2,6,6-tetramethylpiperidine-1-oxyl (TEMPOL), a ROS-scavenging drug, were also orally administered to mice at doses of 300 mg/(kg·d) and 42.5 mg/(kg·d), respectively. Normal saline was administered to age-matched SAMR1 mice, which were used as the normal aging model. During the experiment, the animals’ weights and tail-cuff blood pressures were measured every seven days. Behavioral tests were conducted 30 min after drug administration. The Morris water maze test and object recognition test were performed by all the mice every seven days following drug administration. After four weeks, the mice were euthanized by decapitation. Blood samples were collected from the heart, and the brains were quickly removed and separated into two parts. The cerebral cortex and hippocampus were dissected quickly on ice from one part of the brains and entire brains from the other part were fixed in 10% formalin solution for cresyl violet staining. The vital organs, such as the liver, kidney, spleen, heart, lung, and testicle, were also fixed in 10% formalin solution for hematoxylin and eosin staining. Blood was then immediately centrifuged at 3,000 rpm for 10 min at 4°C to separate the sera. All the sera and brain tissues were stored at −80°C until analysis. Please see the supporting information for detailed methods of the Morris water maze test, object recognition test, measurement of the density of suviving neurons in the brain, antioxidant enzyme, ROS products, cytokine product, body weight, organ weight, blood pressure, hepatic function, acetylcholinesterase (AChE) activity assays, and histopathology.

### Statistical analysis

All data are presented as the mean ± SEM values. The intergroup differences in the latency time in the Morris water maze test and exploration time in the object recognition test were analyzed by two-way analysis of variance (ANOVA) with repeated measurements. The other data were analyzed by one-way ANOVA followed by the Tukey’s post hoc test. A *P* value of less than 0.05 was considered significant. Statistical analysis was performed using the SPSS 17.0 software package for Windows.

## Results

### Delivery of the redox polymer to the brain by oral administration of RNP^N^

**Fig 1:**
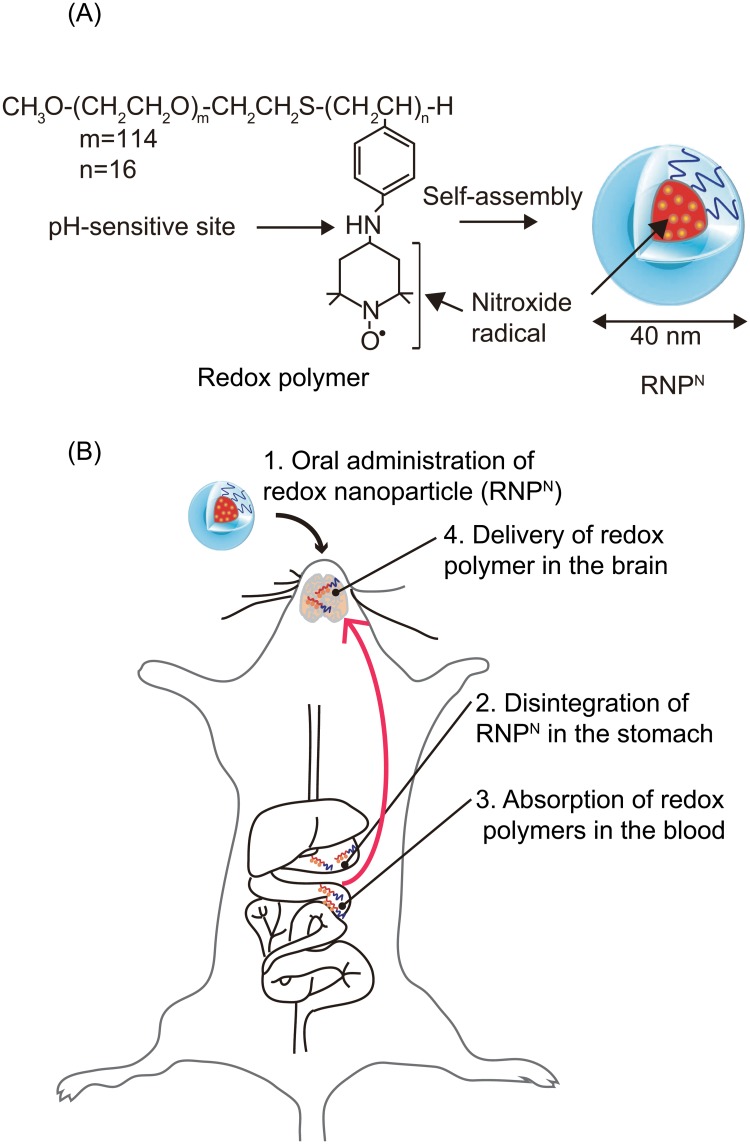
Concept of orally administered redox polymer nanotherapeutics for treatment of the senescence-accelerated neurodegenerative diseases. (A) Structures of redox polymers and RNP^N^ and (B) illustration of delivery of redox polymer to the brain after oral administration of RNP^N^ are shown.

**Fig 2:**
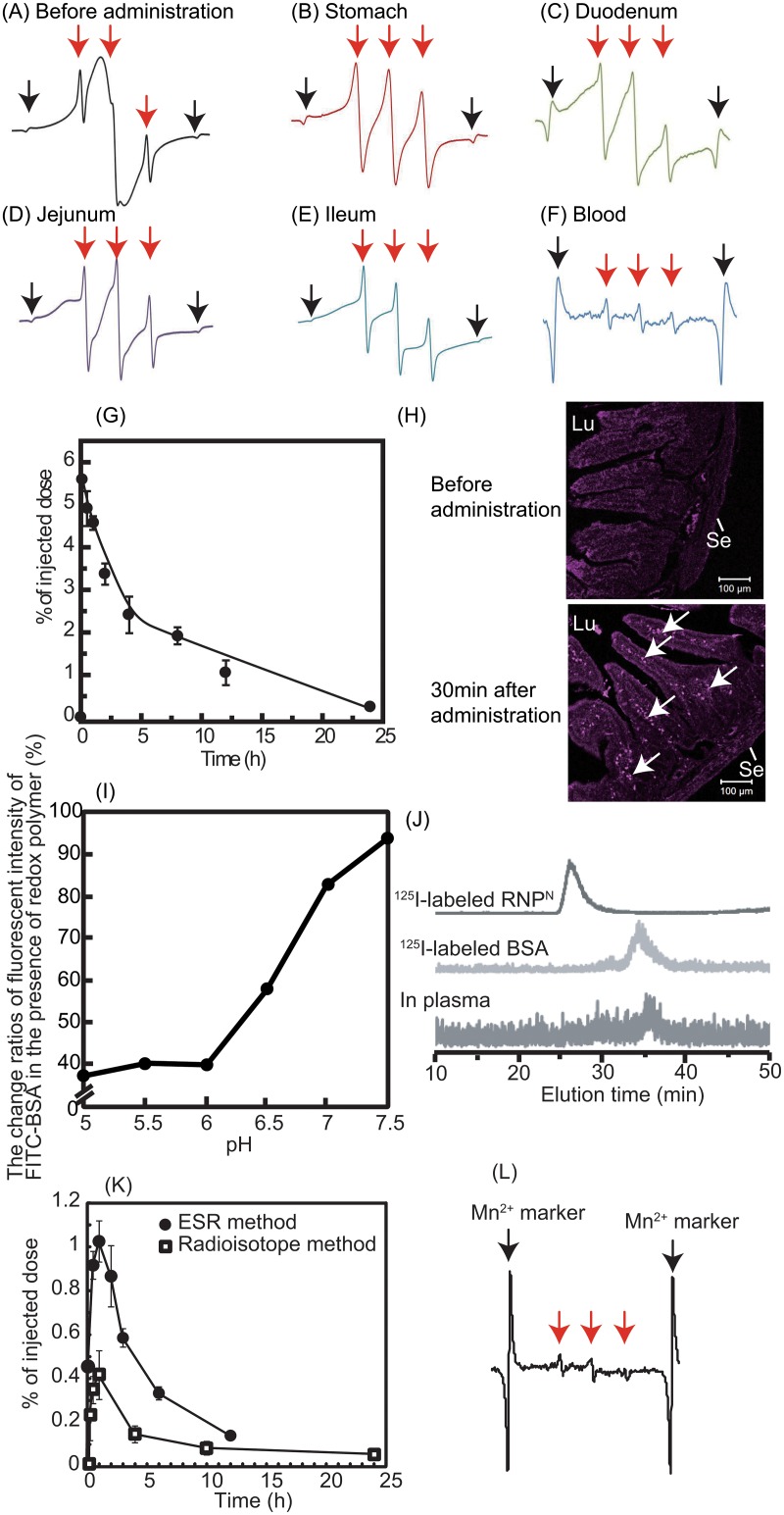
Delivery of redox polymers to the brain after oral administration of RNP^N^ (A) An ESR spectrum of RNP^N^ before its oral administration is shown. (B-F) At 30 min after oral administration of RNP^N^ (300 mg/kg), ESR spectra in (B) the stomach, (C) the duodenum (D) the jejunum, (E) the ileum, and (F) the blood are shown. Red arrows indicate the ESR signal of RNP^N^ or redox polymers. Black arrows indicate the ESR signal of Mn^2+^ marker. (G) The biodistribution of RNP^N^ was determined using ^125^I-labeled RNP^N^. The percentage of radioactivity in the blood was determined by comparison to the injected total radioactivity. The data are expressed as the mean ± SEM values (n = 5). (H) The localization of redox polymer in the duodenum was determined after oral administration of Cy5.5-labeled RNP^N^. Mice were sacrificed at 0.5 h after oral administration of 1 mL of Cy5.5-labeled RNP^N^ at a dose of 2 mg/mL, and the duodenum section was cut circularly. The localization of Cy5.5-labeled redox polymer in the duodenum was analyzed by fluorescent confocal microscopy (Zeiss LSM 700 under oil immersion; Scale bars = 100 μm). Lu and Se in the figure indicate lumen and serosa, respectively. Arrows indicate fluorescent signal of Cy5.5-labeled redox polymer. (I, J) Redox polymers interacted with serum proteins in the bloodstream after oral administration. (I) Interaction between redox polymers and FITC-BSA *in vitro*, determined by fluorescent quenching of FITC-BSA by nitroxide radical moieties in redox polymers (n = 1). (J) Interaction between redox polymers with serum proteins in the blood. Chromatogram of ^125^I-labeled RNP^N^ (upper chromatogram), ^125^I-labeled BSA (middle chromatogram), and the blood sample after oral administration of ^125^I-labeled RNP^N^ (lower chromatogram). (K) The biodistribution of RNP^N^ in the brain via oral administration using ^125^I-labeled RNP^N^ (white square) and ESR measurement (black circle). The data are expressed as mean ± SEM, n = 5. (L) ESR spectrum of redox polymer in the brain at 30 min after oral administration of RNP^N^ (500 mg/kg). Red arrows show the ESR signal of redox polymers. Black arrows show the ESR signal of Mn^2+^ marker.

RNP^N^ with an average diameter of about 40 nm (polydispersity factor, μ/*Γ* ^2^ = 0.04) was prepared by self-assembly of the synthesized redox polymer via the dialysis method (see Fig 1A). First, to confirm the brain delivery of redox polymer after oral administration of RNP^N^ via the pathway as shown in Fig 1B, we measured electron spin resonance (ESR) signal of redox polymers in the stomach, small intestine and blood. ESR spectra of nitroxide radicals in the redox polymer provide information about the morphological state, in which a broad ESR signal indicates the formation of polymeric micelles, and a triplet ESR signal indicates the molecularly dissolved state of redox polymers [20]. As shown in Fig 2A–2F, we measured ESR spectra in the stomach, duodenum, jejunum, ileum, and blood after oral administration of RNP^N^. The ESR signal of RNP^N^ in the vial (before administration) was broad, indicating confinement of nitroxide radicals in the solid core of polymeric micelles (see Fig 2A). After oral administration of RNP^N^, a sharp triplet ESR signal was observed in the stomach, indicating that the disintegration of RNP^N^ in the stomach and exposure of nitroxide radical from the core of RNP^N^ occurred owing to the acidic microenvironment (see Fig 2B). As shown in Fig 2C–2E, even in the duodenum, jejunum, and ileum, ESR spectra of the redox polymer showed triplet signals, demonstrating that redox polymers do not form micelles under these conditions. As shown in Fig 2F, we observed a triplet ESR spectrum of redox polymer in the bloodstream, indicating that the redox polymer was absorbed into bloodstream. The blood uptake of orally administered redox polymers was evaluated by radioisotope measurements. As seen in Fig 2G, 5–6% of the injected dose of the redox polymers was absorbed into the bloodstream and circulated over 24 h. Previously, we confirmed that orally administered TEMPOL is eliminated within 1 h from the blood [7], which is in sharp contrast to orally administered RNP^N^. To get information on whether redox polymers are absorbed across the intestinal epithelium, Cy5.5-labeled RNP^N^ was orally administered, and intestinal sections were observed by fluorescent confocal microscopy. As shown in Fig 2H, strong fluorescent signals were observed inside the villi of the intestinal tissues after oral administration of Cy5.5-labeled RNP^N^. This fluorescent signal was observed over 12 h (see Fig A in S1 File). We have previously confirmed that pH-insensitive redox nanoparticles (RNP^O^, which are not disintegrated in any area of the gastrointestinal tract) maintained a micellar form in the stomach and intenstine. Actually, after oral administration, RNP^O^ accumulated in the surface of the intestinal epithelium, but not inside the villi, because diffusion of RNP^O^ across the intestinal mucus layer to reach the epithelium is difficult, given its size of 40 nm [7, 21]. Contrary to the pH-insensitive RNP^O^, the redox polymer after disintegration of RNP^N^ was internalized deeply in the villi across the intestinal epithelium. Considering both the ESR spectra shown in Fig 2F and the fluoresceint signal of Cy5.5-labeled redox polymer in the villi shown in Fig 2H, it seems that the redox polymers were absorbed in the bloodstream across the intestinal epithelium. It should be noted that cleaved LMW nitroxide radicals and/or Cy5.5 did not contribute to these results because they were covalently conjugated to the polymer. After internalization of the redox polymer into the bloodstream, its cationic charge of the redox polymer might play an important role in interaction with blood proteins. In order to confirm the interaction between the redox polymer and albumin, which is one of the negatively charged proteins in the blood, model experiments were carried out using fluorescent-labeled albumin. Fig 2I shows the change in fluorescent intensity of fluorescein-labeled bovine serum albumin (BSA) in the presence of the redox polymers as a function of pH. Under neutral conditions, almost no change in fluorescent intensity based on the fluorescein-labeled BSA was observed in the presence of redox polymers, which form a core—shell-type RNP^N^ at neutral pH, while a dramatic decrease in fluorescent intensity of fluorescein-labeled BSA was observed with decreasing pH. It is known that nitroxide radicals quench fluorescence when they are in proximity of each other [22]. The observed decrease in the fluorescent intensity means that fluorescent quenching occurred between the fluorescein on albumin and the nitroxide radicals of redox polymers by disintegration of RNP^N^ at the acidic pH, indicating that the redox polymer itself interacts with albumin. The interaction between the blood proteins and the redox polymers also was confirmed *in vivo* by size exclusion chromatography using a radioisotope detector. As shown in Fig 2J, ^125^I-labeled RNP^N^ had an elution time of 27 min, while the elution time of ^125^I-labeled BSA was 35 min; the lowest chromatogram was obtained from mouse serum at 2 h after oral administration of ^125^I-labeled RNP^N^. A low level, but definite peak, was observed at approximately 36 min, which provides some evidence that the absorbed redox polymers interact with blood proteins and circulate over a long period, although the molecular weight of each polymer was 10 kDa. On the basis of these data, it was concluded that the long-term circulation of redox polymers was achieved by the formation of a complex between the cationic redox polymers and blood proteins following gradual internalization of the redox polymers through the intestinal epithelium. Next, the uptake of orally administered RNP^N^ to the brain of normal mice was investigated. As shown in Fig 2K, the uptake of orally administered RNP^N^ in the brain was measured, both by ^125^I-labeled RNP^N^ and by ESR analyses. Small, but definite signals, were observed (0.5–1.0% of injected dose) several hours after oral administration of the RNP^N^. In our previous study, we found that cationic large molecular weight compounds avoid P-glycoprotein pathway due to the large size and preferable internalizes via endocytosis pathway [23]. Since MeO-PEG-*b*-PMNT also possesses cationic PMNT segment, it might avoid drug efflux by P-glycoprotein and be internalized in the brain via endocytosis pathway. In addition, the long-term access of our redox polymers coupled with serum protein to the brain vessel wall by extended blood circulation tendency might increase internalization tendency to the brain. Since redox catalytic species are covalently conjugated to redox polymers as stated above, they were internalized together with the polymers. Fig 2L shows the triplet ESR signal of redox polymer in the normal brain after oral administration of RNP^N^, which is strong proof of the delivery of the redox polymer to the brain. Previous studies reported that SAMP8 mice was found to increase permeability of blood-brain-barrier (BBB) [24] and also show P-glycoprotein deficiency [25]. Thus, we assume that the redox catalytic species could be internalized into the brain of SAMP8 mice.

### Effect of redox polymers in a mouse model of age-associated deficiency in learning and memory

**Fig 3:**
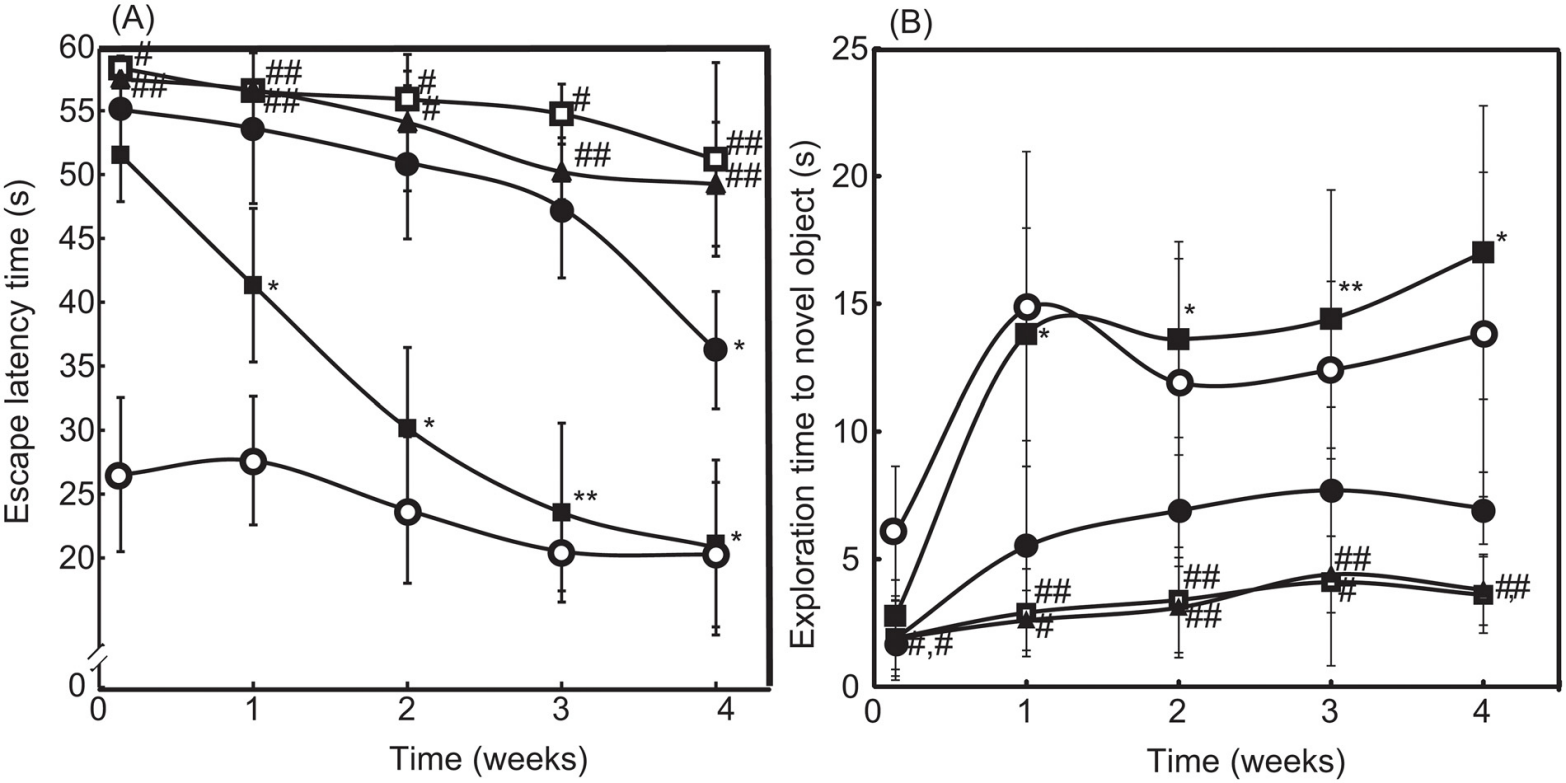
Therapeutic effect of RNP^N^ on cognitive dysfunction. (A) The latency periods of saline-treated SAMR1 mice (open circle), saline-treated SAMP8 mice (open square), blank micelles-treated SAMP8 mice (closed triangle), TEMPOL-treated SAMP8 mice (closed circle), and RNP^N^-treated SAMP8 mice (closed square) were measured by the Morris water-maze test. The values are expressed as mean ± SEM values (n = 10). ^#^P < 0.05, ^##^P < 0.01, compared with SAMR1 mice. *P < 0.05, **P < 0.01, compared with SAMP8 control mice. (B) The exploration times of saline-treated SAMR1 mice (open circle), saline-treated SAMP8 mice (open square), blank micelles-treated SAMP8 mice (closed triangle), TEMPOL-treated SAMP8 mice (closed circle), and RNP^N^-treated SAMP8 mice (closed square) were measured by the object-recognition test. The values are expressed as mean ± SEM values (n = 10). ^#^P < 0.05, ^##^P < 0.01, compared with SAMR1 mice. *P < 0.05, **P < 0.01, compared with SAMP8 control mice.

Here, SAMP8 mice were used as a model of age-associated deficiency in learning and memory [26]. The latency period in the Morris water maze test and the exploration time for obtaining novel objects in the object-recognition test were assessed once per week for one month, as shown in Fig 3A and 3B, respectively. Compared with SAMR1 mice with normal aging characteristics (see S1 Video), the SAMP8 mice required a longer latency period and showed lesser novel exploration time (P < 0.01 for both) owing to the learning and memory deficits (see S2 Video). After treatment for four weeks, the symptoms did not improve significantly, even after the administration of LMW TEMPOL. In contrast, oral administration of RNP^N^ led to significant recovery of the symptoms (see S3 Video). As can be seen in Fig 3A, four weeks of treatment with RNP^N^ drastically decreased in escape latency time from 51.68 s to 20.96 s, which reached same level as SAMR1 mice (20.4 s at four weeks). In addition, surprisingly, after two weeks of treatment, the RNP^N^-treated group had evidently higher novel exploration times than those of the saline-treated group; exploration times of RNP^N^-treated SAMP8 and SAMR1 mice are 17 s and 13 s at four weeks, respectively (see Fig 3B). These results demonstrated that the redox polymers were effective in improving the learning ability of SAMP8 mice.

**Fig 4:**
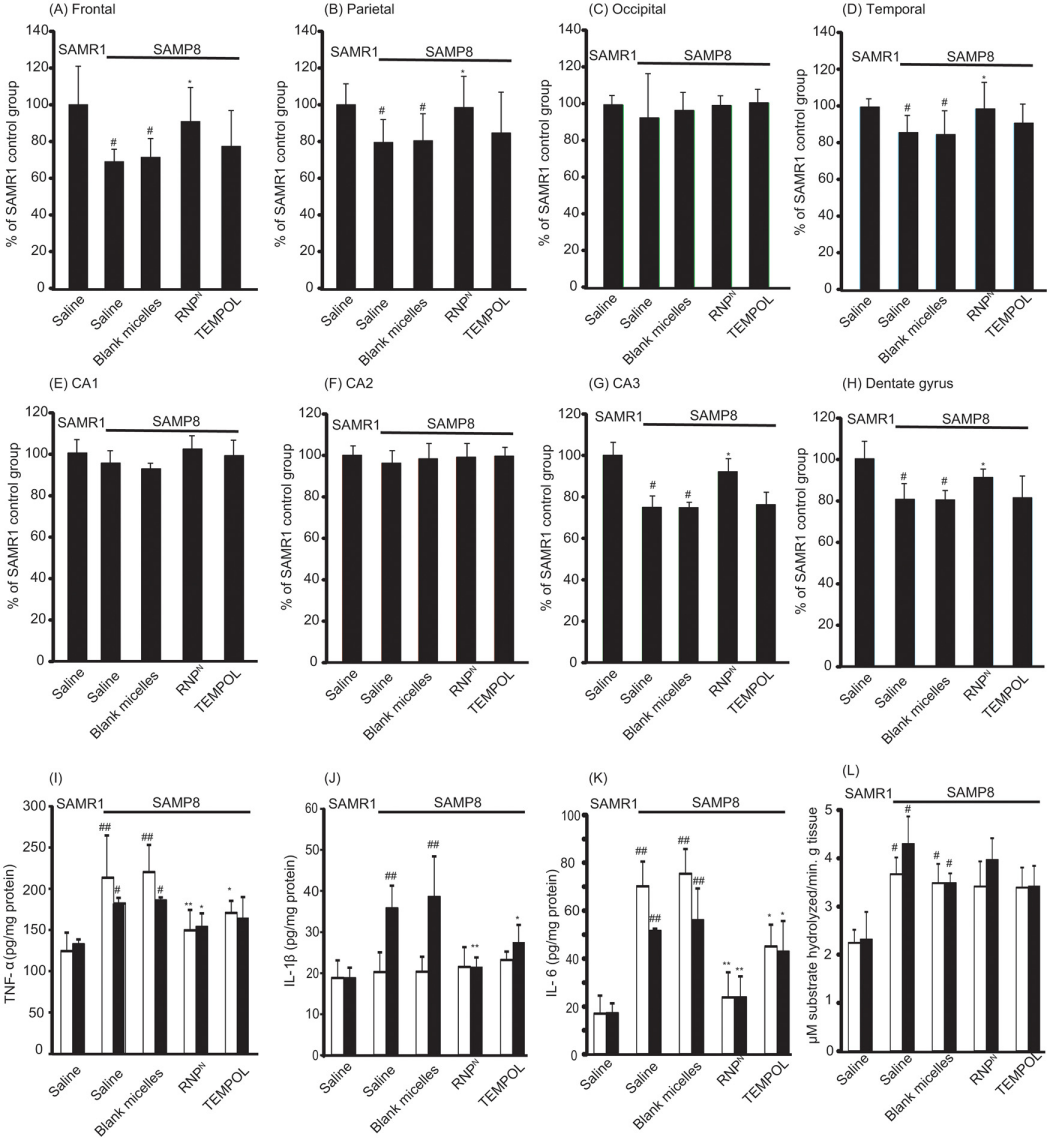
The density of surviving neurons in SAMP8 mice was increased by oral administration of RNP^N^. The densities of surviving neurons in various subregions of (A) frontal, (B) parietal, (C) occipital, (D) temporal, (E) CA1, (F) CA2, (G) CA3, and (H) dentate gyrus in the brain of SAMP8 mice were assessed by cresyl violet staining. The values are expressed as the mean ± SEM values (n = 5). ^#^P < 0.05, compared with SAMR1 mice. *P < 0.05, compared with SAMP8 control mice. (I-K) Levels of proinflammatory cytokines of (I) TNF-α, (J) IL-1β and (K) IL-6 in the cortex (white bar) and hippocampus (black bar) areas of the brain of SAMR1 and SAMP8 mice are shown. Values are expressed as mean ± SEM (n = 10). ^#^P < 0.05, ^##^P < 0.01, compared with SAMR1 mice. *P < 0.05, compared with SAMP8 control mice. (L) Effect of redox polymer nanotherapeutics on acetylcholinesterase (AChE) activity in the SAMP8 mice brain. Values are expressed as mean ± SEM (n = 10). ^#^P < 0.05 compared with SAMR1 mice.

We determined the effect of RNP^N^ on neuron density in various sub-regions of the cortex and the hippocampal areas, which play important roles in learning and memory. It was found that, in the brain of SAMP8 mice, neuron densities significantly decreased in the frontal, parietal, and temporal areas of the cortex and in the CA3 and dentate gyrus of the hippocampus (P < 0.05 for all). It is interesting to note that the neuron densities of the RNP^N^-treated SAMP8 mice were almost the same as those of the SAMR1 mice, as shown in Fig 4A–4H. The results clearly demonstrated that the redox polymer significantly attenuated neurodegeneration.

To evaluate the oxidative stress state in the brain of SAMP8 mice, we measured the levels of malondialdehyde (MDA), protein carbonyl, 8-hydroxy-2′-deoxyguanosine (8-OHdG), and nitric oxide (NO), as well as superoxide scavenging activities (% inhibition of superoxide anion) and the levels of SOD, GPx, and CAT, in the brain after treatment. As shown in Tables 1 and 2, MDA, protein carbonyl, 8-OHdG, and NO were significantly higher in the brains of SAMP8 mice than those of SAMR1 mice (P < 0.01 for all). Treatment with redox polymer significantly decreased levels of MDA, protein carbonyl, 8-OHdG, and NO, compared with SAMP8 control mice (P < 0.01 for all). Compared with SAMR1 mice, SAMP8 mice showed a significant decrease of 86.9% (P < 0.01), 16.6% (P < 0.05), and 21.3% (P < 0.05) in the activities of SOD, CAT, and GPx in the cortex area of the brain, respectively. Treatment with redox polymer markedly increased only SOD activity in the brain of SAMP8 mice (P < 0.01). In fact, the activities of superoxide scavenging in the brain after treatment with redox polymers are higher than those of TEMPOL-treated mice. In addition, levels of inflammatory cytokines in the brain after one month of treatment were measured. Compared with SAMR1 mice, SAMP8 mice have higher levels of the pro-inflammatory cytokines, TNF-α, IL-1β, and IL-6, especially in the hippocampus area, as shown in Fig 4I–4K, respectively. Treatment with redox polymer decreased the levels of those pro-inflammatory cytokines. Since changes within the cholinergic systems so far have been reported to be involved in cognitive and behavioral functions that are widely disturbed in AD [27], we evaluated the effects of redox polymers on AChE activity in SAMP8 mice. As shown in Fig 4L, however, we did not find the amelioration of AChE activity in the cortex and hippocampus areas by treatment with redox polymer, indicating that the therapeutic effect of oral administration of RNP^N^ is attributable to the suppression of oxidative stress, but not the AChE pathway.

**Table 1:** The levels of MDA, protein carbonyl, 8-OHdG, NO, superoxide scavenging activity (% inhibition of superoxide anion), antioxidant enzyme activity of SOD, catalase and GPx in the cortex area of SAMP8 mice.

Animal	SAMR1 mice	SAMP8 mice			
Administered drug	Saline	Saline	Blank micelles	RNP^N^	TEMPOL
MDA (μM/μg protein)	0.037 ± 0.06	0.114 ± 0.029^##^	0.106 ± 0.041^##^	0.040 ± 0.020**	0.075 ± 0.025*
protein carbonyl (nM/mg protein)	0.718 ± 0.090	1.483 ± 0.275^##^	1.648 ± 0.256^##^	0.820 ± 0.111**	1.122 ± 0.321*
8-OHdG (nM/mg protein)	6.950 ± 1.614	15.441 ± 2.567^##^	14.270 ± 1.985^##^	11.284 ± 3.775**	10.856 ± 1.366**
SOD (units/mg protein)	1.025 ± 0.119	0.134 ± 0.027^##^	0.113 ± 0.04^##^	0.852 ± 0.04**	0.335 ± 0.06*
CAT (units/mg protein)	7.583 ± 0.960	6.325 ± 0.823^#^	5.695 ± 0.536^#^	6.115 ± 0.378	7.116 ± 1.659*
GPx (units/mg protein)	18.754 ± 1.904	14.755 ± 1.392^#^	13.924 ± 1.730^#^	14.177 ± 1.275	11.720 ± 0.747
NO (μM/g tissue)	18.387 ± 3.754	47.081 ± 5.551^##^	45.121 ± 3.483^##^	30.664 ± 9.534**	39.571 ± 4.613*
superoxide anion (% inhibition/mg tissue)	63.878 ± 4.61	17.827 ± 6.427^##^	15.326 ± 4.896^##^	54.021 ± 6.751**	37.133 ± 8.532**

(n = 5)

^#^P < 0.05

^##^P < 0.01, compared with SAMR1 mice

*P < 0.05

**P < 0.01, compared with SAMP8 control mice

**Table 2:** The levels of MDA, protein carbonyl, 8-OHdG, NO, superoxide scavenging activity (% inhibition of superoxide anion), antioxidant enzyme activity of SOD, catalase and GPx in the hippocampus area of SAMP8 mice.

Animal	SAMR1 mice	SAMP8 mice			
Administered drug	Saline	Saline	Blank micelles	RNP^N^	TEMPOL
MDA (μM/μg protein)	0.034 ± 0.002	0.082 ± 0.011^##^	0.074 ± 0.008^##^	0.036 ± 0.002**	0.053 ± 0.010**
protein carbonyl (nM/mg protein)	0.771 ± 0.100	1.166 ± 0.339^##^	1.078 ± 0.130^##^	1.007 ± 0.092**	0.867 ± 0.093
8-OHdG (nM/mg protein)	10.081 ± 2.993	23.421 ± 3.236^##^	24.778 ± 4.193^##^	12.940 ± 2.254**	14.824 ± 1.267**
SOD (units/mg protein)	1.644 ± 0.343	0.642 ± 0.099^##^	0.325 ± 0.063^##^	1.445 ± 0.284**	0.830 ± 0.155*
CAT (units/mg protein)	7.682 ± 1.162	7.985 ± 0.233	7.116 ± 0.690	7.851 ± 0.428	7.107 ± 0.596
GPx (units/mg protein)	21.108 ± 3.297	16.265 ± 2.186^#^	14.614 ± 2.310^#^	15.950 ± 2.159	15.719 ± 1.060
NO (μM/g tissue)	21.858 ± 5.692	48.263 ± 4.385^##^	46.384 ± 3.023^##^	35.230 ± 3.047**	40.873 ± 7.587*
superoxide anion (% inhibition/mg tissue)	63.229 ± 4.468	19.075 ± 2.989^##^	21.712 ± 12.526^##^	46.662 ± 5.869**	36.900 ± 9.592**

(n = 5)

^#^P < 0.05

^##^P < 0.01, compared with SAMR1 mice

*P < 0.05

**P < 0.01, compared with SAMP8 control mice

### Toxicity of orally administered RNP^N^ for treatment of senescence-accelerated prone mice

**Fig 5:**
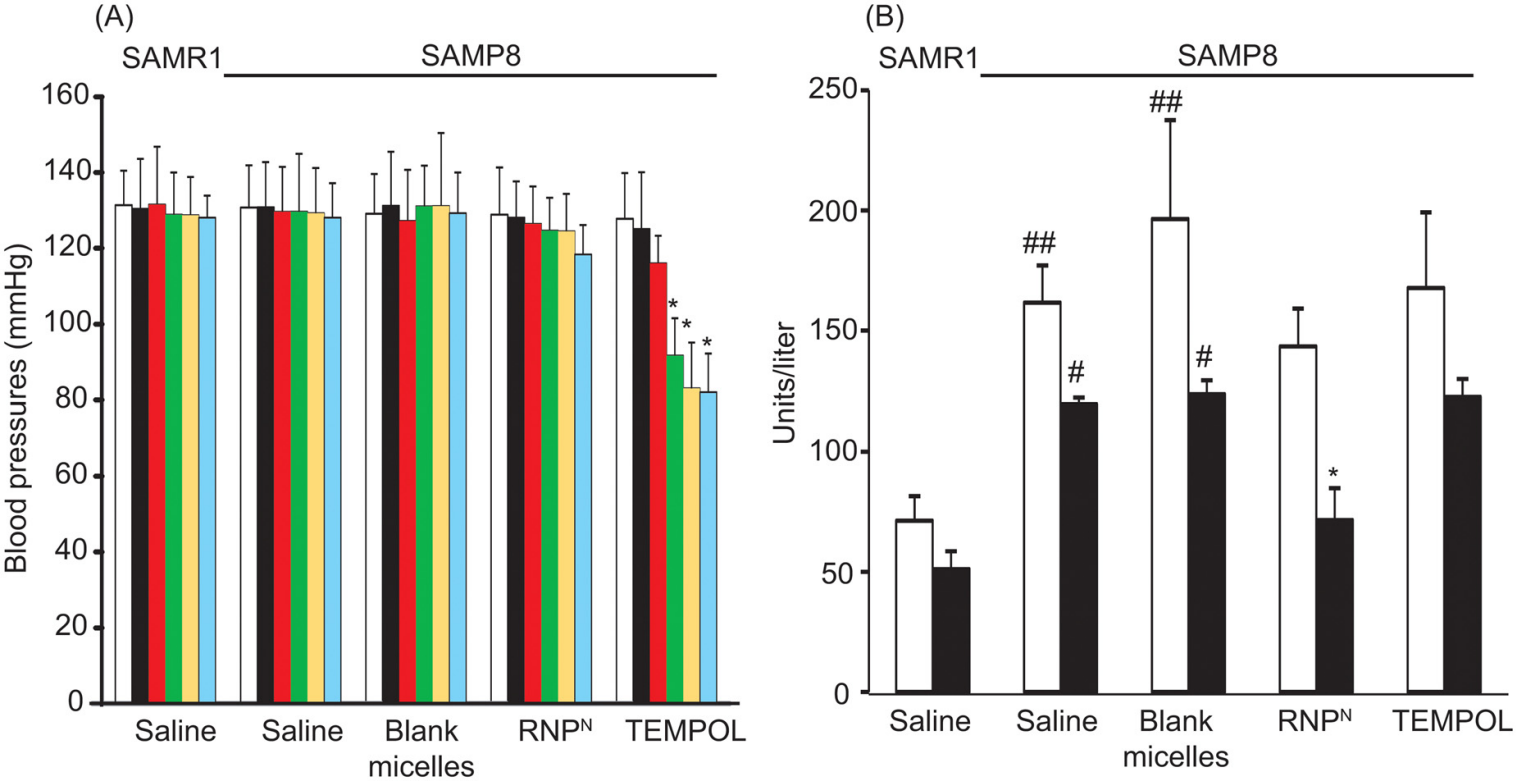
Measurement of adverse effects. (A) Effects of RNP^N^ on blood pressure of SAMP8 mice in tail-cuff blood pressure method. Blood pressures before administration (white bar), after single administration (black bar), one week after starting treatment (red bar), two weeks after starting treatment (green bar), three weeks after starting treatment (yellow bar), and four weeks after starting treatment (blue bar) are shown. Values are expressed as mean ± SEM (n = 10). *P < 0.05 compared with SAMP8 control mice. (B) Effects of redox polymer nanotherapeutics on AST (white bar) and ALT (black bar) levels of SAMP8 mice. Values are expressed as mean ± SEM (n = 10). ^#^P < 0.05, ^##^P < 0.01 compared with SAMR1 mice. *P < 0.05 compared with SAMP8 control mice.

To assess the safety of redox polymer nanotherapeutics, we measured the mouse body weight, vital organ weight, hepatic function including aspartate amino transferase (AST) and alanine transaminase (ALT) levels, blood pressure, and the histological features of vital organs by hematoxylin and eosin staining. Before explanation of these evaluations, it should be noted that all mice died when hydrophobic TEMPO was administered under the same dose as other experiments. As stated above, internalization of hydrophobic TEMPO in healthy cells disturbed normal redox reactions, especially electron transport chain, to cause severe adverse effects. Even oral administration of hydrophilic TEMPOL significantly reduced blood pressure in the SAMP8 mice after two weeks, as shown in Fig 5A. It is interesting to note that treatment with redox polymer did not show this adverse effect. In addition, no deaths were observed throughout the experimental period, which is in sharp contrast to LMW TEMPOL. There were no significant differences in the weights of body and major organs (liver, spleen, kidney, lung, testicle and heart) of animals in all the treatment groups (see Table A in S1 File). Gross pathological changes due to administration of any of the substances were not observed in any of the organs. Histopathological examination of the organs did not show any abnormalities and conspicuous damages in tissues (see Fig B in S1 File). Regarding hepatic function, as shown in Fig 5B, it was found that SAMP8 mice showed higher serum levels of both AST and ALT than those of SAMR1 mice (P < 0.01 and P < 0.05, respectively), which corresponds to a previous report [28]. When RNP^N^ was orally administered to SAMP8 mice, these levels tended to decrease (P < 0.05 in the level of ALT), which is promising in terms of liver protection. In our current study, we have confirmed that orally administered RNP^N^ shows therapeutic effect of non-alcoholic steatohepatitis because redox polymers are delivered to the liver (Eguchi et al., submitted for publication).

## Discussion

In this study, we newly developed oral redox polymer nanotherapeutics for treatment of chronic diseases. It was confirmed that the redox polymer was absorbed into the blood after disintegration of the nanoparticle in the stomach through the intestinal epithelium as expected, followed by delivery of redox polymer to the brain. The oral administration of RNP^N^ to the SAMP8 mice for one month exhibited an improvement of cognitive function without adverse effects by suppressing oxidative stress in the brain, unlike treatment with LMW TEMPOL.

One of the important characteristics of redox polymer nanotherapeutics is long-term circulation of redox polymer in the bloodstream after oral administration of RNP^N^. The brain delivery of nanoparticle with ability of long-term blood circulation has been so far reported by several research groups using normal mice [29–32]. Similar to the EPR effect, the continuous access of the nanoparticles to the cerebral blood vessels due to long-term blood circulation might increase its uptake in the brain due to the fairly large access area of cerebral blood vessel walls. In fact, we could confirm internalization of the redox polymers even in brain of normal mice, which indicates that this continuous access tendency might work well (see Fig 2K and 2L). In addition, since it was reported that aged SAMP8 mice have vessels with a disrupted BBB and even IgG with 150 kDa are internalized in the brain, more redox polymers might be delivered to the brain of SAMP8 mice [24]. Although, for treatment of brain deseases, intravenously [33] and intranasally [34] administered nanomedicines have been reported, there are no reports of orally administered nanomedicine. Moreover, the LMW anti-oxidative therapies failed due to its easily internalize in healthy cells and disruption of normal redox reaction such as electron transport chain. This limits application using enough amount of LMW drug administration. Here, we emphasize that this redox polymer nanotherapeutics is the first concept of orally injectable nanomedicine using the pH-responsiveness of redox polymers. In addition, since redox polymers suppress the systemic oxidative stress after oral administration of RNP^N^, it must possess the potential to decrease the risk of various oxidative stress-related diseases.

## Conclusions

In conclusion, we demonstrate that orally administered pH-sensitive redox nanoparticles almost completely revived the cognition in 17-week-old SAMP8 mice (Fig 3A and 3B). Small, but evident, amounts of the redox polymer were internalized in the brain of normal mice (as shown in Fig 2K and 2L). After treatment with redox nanoparticles, ROS levels were decreased significantly in the brain of SAMP8 mice (as shown in Tables 1 and 2), which is probably due to the long access of redox polymers to blood vessel in brain. The scavenging of ROS in the brain prevented oxidative stress and resulted in recovery of endogenous anti-oxidative enzymes (Tables 1 and 2), thus protecting neuronal cells effectively (Fig 4A–4H). In addition, orally administered redox polymers did not show any detectable toxicity to main organs (Fig 5A and 5B and Fig B and Table A in S1 File).

## Supporting Information

S1 File Text of further experimental procedures, Fig A and B, and Table A. (DOCX)

S1 Video The Morris water-maze test of SAMR1 mice. (MOV)

S2 Video The Morris water-maze test of SAMP8 mice. (MOV)

S3 Video The Morris water-maze test of RNP^N^-treated SAMP8 mice. (MOV)
