# Supplementary material for: Recovery of Cognitive Dysfunction via Orally Administered Redox-Polymer Nanotherapeutics in SAMP8 Mice
Source: PLoS One. 2015 May 8;10(5):e0126013. doi: 10.1371/journal.pone.0126013 (PMC4425673; doi:10.1371/journal.pone.0126013)
Supplement: S1 File — (DOCX) [file pone.0126013.s001.docx]

**S1 File**

**Recovery of Cognitive Dysfunction via Orally Administered Redox-polymer Nanotherapeutics in SAMP8 Mice**

Pennapa Chonpathompikunlert, Toru Yoshitomi, Long Binh Vong, Natsuka Imaizumi, Yuki Ozaki, Yukio Nagasaki

**Preparation of the RNP^N^**

The RNP^N^ was prepared by the self-assembly of MeO-PEG-*b*-PMNT (MW [PEG] = 5,500 Da; MW [PMNT] = 4,500 Da) using the dialysis method reported previously [1, 2]. Briefly, 300 mg of MeO-PEG-*b*-PMNT was dissolved in 3 mL of dimethylformamide (DMF). The polymer solution was transferred into a membrane tube (Spectra/Por; molecular-weight cutoff size: 3,500 Da; Spectrum, Houston, TX, USA) and then dialyzed for 24 h against 2 L of water, which was changed after 2, 5, 8, 12, and 20 h. Dynamic light scattering (DLS) measurements were carried out to determine the diameter of the RNP^N^ obtained after dialysis. In order to adjust the concentration of the RNP^N^ solution, the solution was concentrated by a centrifugal evaporator (~ 60 mg/mL), followed by DLS measurements of the concentrated RNP^N^ to confirm the size of the RNP^N^ after the concentration step. At more than 60 mg/mL, viscosity of RNP^N^ solution is increased. Since high viscosity of RNP^N^ solution makes injection more difficult, 60 mg/mL of RNP^N^ concentration was used in this study. Electron spin resonance (ESR) signals were measured to confirm the amount of nitroxide radical inside the nanoparticle.

**Preparation of ^125^I-labeled RNP^N^**

A solution of Na^125^I in 10 mM phosphate-buffered saline (PBS) (100 μL, 74 MBq/mL, PerkinElmer, Inc., Wellesley, MA, USA) was added to a solution of RNP^N^ (2mL, 50 mg/mL). A solution of chloramine T (40 μL, 600 mM, Sigma-Aldrich, Tokyo, Japan) in 10 mM PBS was added to the reaction mixture, which was incubated at room temperature for 10 min. After incubation, the unreacted ^125^I and other chemicals were removed by three rounds of centrifugation device (3,200 rpm) using a membrane ultrafiltration (Vivaspin, MWCO: 5000, GE Healthcare, Milwaukee, WI, USA). To confirm the purification, gel filtration chromatography was conducted on a PD-10 column (GE Healthcare) using saline as the eluent. The radioactivity of each fraction was measured using a γ-counter (Aloka, Japan). The^125^I-labeled albumin was prepared following the same procedure as that for ^125^I-labeled RNP^N^ by using BSA (200 μL, 10 mg/mL, Sigma-Aldrich), Na^125^I (2 μL) and chloramine T (10 μL, 600 mM).

**Pharmacokinetics of redox polymer by ^125^I-labeled RNP^N^**

The mice were fasted 1 d before the experiment, and then 0.5 mL of ^125^I-labeled RNP^N^ (20 mg/mL) was orally administered to ICR mice. The mice were subsequently sacrificed at 0.25, 0.5, 1, 2, 4, 8, 10, 12, and 24 h after oral administration. After sacrifice, blood samples were obtained from the heart, and brain samples were isolated. Radioactivity of the samples was measured by a γ-counter (ARC-380; Aloka, Japan). The percentage of radioactivity in each organ was determined based on the injected total radioactivity.

**ESR measurement after oral administration of RNP^N^**

RNP^N^ (300 mg/kg) was orally administered to ICR mice (body weight; 30 g). Then, ICR mice were anesthetized via an intraperitoneal injection of pentobarbital sodium (40 mg/kg). To measure the concentration of redox polymer in the brain, the brain tissues were excised at 0.083, 0.5, 1, 2, 3, 6, and 12 h after the administration. For measurement of ESR spectra, tissues of the stomach, duodenum, jejunum, ileum, blood, and brain samples were obtained at 30 min after oral administration of RNP^N^. The isolated tissues were immediately placed on ice. The tissue homogenates were prepared by a homogenizer, except for blood. Blood was collected by cardiopuncture using heparinized syringes. The ESR spectra were measured by X-band ESR measurements (JES-TE25X; JEOL, Tokyo, Japan) after addition of 10 mM K_3_[Fe(CN)_6_], which was prepared at 200 mM as a stock solution. The ESR measurements were carried out under the following conditions: frequency, 9.41 GHz; power, 8.00 mW; ﬁeld, 333.8 ± 5 mT; sweep time, 1.0 min; modulation, 0.1 mT; time constant, 0.1 s. Note that the dose of RNP^N^ was 500 mg/kg in the results of ESR spectra in Fig. 2L because of tiny ESR signals.

**Preparation of Cy5.5-labeled RNP^N^**

The secondary amino groups in the PMNT segment of PEG-*b*-PMNT were labeled by Cy5.5 NHS Ester Mono-reactive (GE Healthcare). Briefly, 1 mg of Cy5.5 NHS Ester Mono-reactive was added to the RNP^N^ solution and stirred for 1 h at room temperature. The mixture was transferred into a membrane tube (Spectra/Por; molecular-weight cutoff size: 3,500 Da; Spectrum) and then dialyzed for 24 h against 2 L of water, followed by freeze-drying.

**Localization of Cy5.5-labeled RNP^N^ in the villi of small intestine**

The localization of orally administered RNP^N^ in the small intestine was investigated by Cy5.5-labeled RNP^N^. One milliliter of Cy5.5-labeled RNP^N^ (2 mg/mL) was orally administered to mice, and the mice were sacrificed at 0.5, 1, and 12 h after oral administration. Residues in the duodenum were gently removed with phosphate buffer (pH 7.4), and 7-μm-thick sections of the duodenum were prepared. Fluorescence images (excitation wavelength = 639 nm, emission wavelength = 669 nm) of Cy5.5-labeled RNP^N^ were acquired using a fluorescent confocal microscope system (Zeiss LSM 700 Carl Zeiss Microscopy GmbH, Jena, Germany).

**Interaction between redox polymers and blood serum proteins**

The ^125^I-labeled RNP^N^ was administered to the ICR mice by oral injection at a dose of 1250 mg/kg body weight. Mice were anesthetized with pentobarbital sodium (40 mg/kg), and blood was collected by cardiac puncture using a heparinized syringe at 2 h post-substance injection. After filtration through a 0.45-µm filter, 100 μL aliquots of ^125^I-labeled RNP^N^, ^125^I-labeled albumin, and the plasma were subjected to size exclusion chromatography analysis using a JASCO HPLC system (JASCO, Tokyo, Japan) equipped with a Bioscan flow-count detector (Bioscan Inc., Washington DC, USA), a TSK gel BioAssist G3SWXL, and TSK gel BioAssist G2SWXL system (TOSOH, Tokyo, Japan), with 10 mM phosphate-buffered saline (pH 7.4) at a flow rate of 0.50 mL/min.

**Interaction between redox polymers and FITC-BSA *in vitro***

We prepared 1 mL of RNP^N^ solution (500 μg/mL) at pH values of 5, 5.5, 6.0, 6.5, 7.0, and 7.5. RNP^N^ solutions were mixed with 1 mL of FITC-labeled BSA solution (5 μg/mL) at pH values of 5, 5.5, 6.0, 6.5, 7.0, and 7.5. After 1 h of mixing, the fluorescent intensities of FITC-BSA were measured (ex: 475 nm, em: 519 nm). After measurement, the change ratios of fluorescent intensity of FITC-BSA in the presence of redox polymers as function of pH were calculated from the equation below:

The change ratios of fluorescent intensity of FITC-BSA in the presence of redox polymers (%) = fluorescence intensity of FITC-BSA with redox polymers / fluorescence intensity of FITC-BSA without redox polymers × 100

**Morris water maze test**

A circular water tank (120 × 50 cm) was filled with 25°C water to a depth of 30 cm; an escape platform 11 cm in diameter was placed in the tank, with the top 1 cm below the water surface. The platform was in the middle of the target quadrant, and its position remained fixed during the experiment. Above the tank, a white floor-to-ceiling cloth curtain was drawn around the pool, and four kinds of black cardboard (circle, triangular, rhombus, and square), serving as spatial cues, were hung equidistantly on the interior of the curtain. Each mouse was trained in 4 rounds of trial continuously for 7 days. When they succeeded, mice were allowed to stay on the platform for 30 s. When the mice failed to find the platform within 60 s, they were assisted by the experimenter and allowed to stay the platform for the same time. Probe trials were performed 1, 7, 14, 21, and 28 days after the last training session.

**Object recognition test**

The object recognition test was performed in a circle open-field apparatus (60 × 50 cm). The objects used in this task were different in shape, color, and texture. The open field and the objects were cleaned between each trial using 70% ethanol to avoid odor trails. Before the experiment day, the animals were allowed to acclimatize to the experimental environment. During habituation, the animals were allowed to freely explore the apparatus without objects for 5 min, once a day for three consecutive days before testing. On the experimental day, animals were submitted to two trials. During the first trial (T1), animals were placed in the area containing two identical objects for an amount of time necessary to spend 15 s exploring these two objects with a limit of 4 min. Any mice that did not explore the objects for 15 s within the 4-min period were excluded from experiments. One hour after exposure to the first trial, the animals were exposed to the second trial (T2). According to this trial, one of the objects presented in the first trial was replaced by a novel object. Animals were placed back in the arena for 3 min, and the total times that the animals spent to explore or directed the nose within 2 cm of the object while looking at, sniffing, or touching the novel object were recorded and recognized as total exploration time upon novel object.

**Measurement of the density of surving neurons in the brain of SAMP8 mice**

Two μm-thick brain sections were prepared from parafﬁn blocks using microtome and stained with 0.5% cresyl violet. Images were taken using an OLYMPUS camera connected to the microscope to examine neuronal density in cortex and hippocampus under 40 × magnification according to the same stereotaxic coordinates. Five coronal sections of each mouse in each group were studied quantitatively. Analyses of neuronal stained images were carried out using Image J^TM^ software [3]. The neuronal counts were made in five adjacent fields, and the mean of neuronal counts was extrapolated as the total number of neurons. All data are represented as % of control group.

**Antioxidant enzyme assays**

Superoxide dismutase (SOD) activity was assayed utilizing the technique of previous study[4] based on the inhibition of the rate of reduction of cytochrome C by the superoxide radical, which was observed at 550 nm. In a 1 mL system mixture, the final concentrations were 50 mM potassium phosphate, 0.1 mM ethylenediaminetetraacetic acid (EDTA), 0.01 mM cytochrome C, 0.05 mM xanthine, 0.005 unit xanthine oxidase, and 1 unit superoxide dismutase solution or sample. The superoxide dismutase solution was used as a standard for enzyme activity. The standard curve was plotted as percentage inhibition against the SOD activity. One unit of activity was defined as the amount of enzyme necessary to inhibit the rate of reduction of cytochrome C by 50% in the coupled system using xanthine-xanthine oxidase at pH 7.8 at 25 °C. The data were presented in units of SOD activity per mg protein.

Catalase (CAT) activity was assayed using a photometric method [5]. The enzyme sample or the standard enzyme solution was allowed to react with hydrogen peroxide for 1 min. The reaction was then stopped by a sulfuric acid solution. Potassium permanganate was added to the mixture and allowed to react with the excess peroxide that was not decomposed by catalase. After the addition of permanganate, the excess permanganate from the reaction with peroxide was determined photometrically at 515 nm. The standard curve was plotted as the absorbance at 515nm against the catalase activity. The data were reported in units of catalase per mg protein.

The glutathione peroxidase (GPx) activity was determined using a previous method [6], in which the activity was measured indirectly by a coupled reaction with glutathione reductase. Oxidized glutathione, produced upon reduction of hydrogen peroxide by glutathione peroxidase, was recycled to its reduced state by glutathione reductase and NADPH. The oxidation of NADPH to NADP^+^ was accompanied by a decrease in absorbance at 340 nm (A340 nm). The rate of decrease in the A340 nm was directly proportional to the glutathione peroxidase activity. The final 1 mL of the system mixture contained 48 mM sodium phosphate, 0.38 mM EDTA, 0.12 mM β-NADPH, 0.95 mM sodium azide, 3.2 units of glutathione reductase, 1 mM glutathione (GSH), 0.02 mM DL-dithiothreitol, 0.0007% H_2_O_2_, and the standard enzyme glutathione peroxidase solution or a homogenate brain sample. The glutathione peroxidase solution was used as the standard for enzyme activity. The standard curve was plotted as the rate of A340 nm per minute against the GPx activity. One unit of activity was defined as the amount of enzyme necessary to catalyze the oxidation by H_2_O_2_ of 1 µmol of GSH to glutathione disulfide (GSSG) per minute at pH 7 at 25 °C. The data were reported in units of GPx per mg protein.

**Reactive oxygen species (ROS) products assays**

Lipid peroxidation (LPO) was measured by determining the concentrations of malonyldialdehyde (MDA) and 4-hydroxyalkenals (HAE), which are used as indicators of lipid peroxidation, were measured by using a commercial assay kit (BIOMOL International, Plymouth Meeting, MA, USA). Each sample was homogenized (Potter-Elvehjem) in a 10-fold volume of ice-cold 20 mM of PBS (pH 7.4) containing 0.5 mM butylated hydroxytoluene to prevent sample oxidation. The homogenized sample was centrifuged at 3000 *g* at 4 °C for 10 min, and a 200 µL aliquot of the supernatant was used to measure MDA plus HAE levels according to the instructions of the manufacturer. Values were standardized to micrograms of protein.

Protein carbonyl, which is the most common product of protein oxidation in biological samples, was measured using a commercial assay kit (Cell Biolabs, Inc., San Diego, CA, USA.). Each sample was homogenized (Potter-Elvehjem) in a 10-fold volume of ice-cold 20 mM of PBS (pH 7.4) containing 1% streptomycin sulfate and incubated for 30 min at room temperature. The nucleic acid precipitates were removed by centrifuging at 6000 *g* for 10 min at 4 °C to avoid erroneous contribution to a higher estimation of the carbonyl content from nucleic acid in the cells. The supernatant was used to measure protein levels according to the instructions of the manufacturer. The obtained values were standardized to milligrams of protein. Deoxyguanosine (dG) is one of the constituents of DNA, and when it is oxidized, it is altered into 8-hydroxy-2'-deoxyguanosine (8-OHdG). 8-OHdG is useful as a general DNA oxidation marker in the body. A commercial assay kit (Wako Pure Chemical Industries, Osaka, Japan) was used for this measurement. Tissue homogenate and supernatant were used to measure 8-OHdG levels according to the instructions of the manufacturer. The values obtained were standardized to milligrams of protein.

**Acetyl cholinesterase (AChE) activity assay**

The activity of AChE was measured according to a developed method [7, 8]. This method employed acetylthiocholine iodide (ATChI) as a synthetic substrate for AChE. ATChI is broken down to thiocholine and acetate by AChE and thiocholine reacts with dithiobisnitrobenzoate (DTNB) to produce a yellow color. The quantity of yellow color, which develops over time, was measured the absorbance at 412︎ nm using a spectrophotometer and used as an indicator of the AChE activity.

**Cytokine product assay**

The amounts of tumor necrosis factor (TNF)-α, interleukin-1 beta (IL-1β), and interleukin-6 (IL-6) levels in the supernatant from brain tissue were measured by a commercial enzyme-linked immunosorbent assay kit (Thermo Scientific, Rockford, USA) according to the manufacturer’s instructions. All determinations were performed in duplicate.

**Histopathology**

Lung, heart, liver, kidney, spleen and testicle were isolated for histopathology. Tissues were ﬁxed in 10% formalin solution. Parafﬁn blocks were prepared after completing the tissue processing in different grades of alcohol and xylene. Lung, heart, liver, kidney, spleen, and testicle sections (thickness: 2 µm) were prepared from parafﬁn blocks using a microtome, and stained with hematoxylin and eosin. Images were taken using an OLYMPUS camera connected to the microscope to examine gross cellular damage

**Assay of superoxide anion scavenging activity of the brain tissue after treatment**

The reaction mixture consisted of 10 mM phosphate buffer (pH 7.4) containing 0.1 mM xanthine, 0.1 mM EDTA, 0.1 mM nitroblue tetrazolium, and 0.1 unit xanthine oxidase at a final volume of 1 mL. The formation rate of formazan produced was determined from the slope of the absorbance curve during the initial 2 min of the reaction at 560 nm [9]. In order to analyze the antioxidant activity, each sample of different groups was added to the reaction mixture. The change in absorbance was compared with that of the control in the same time reaction, and anti-oxidation activity was calculated according to the following equation: anti-oxidation activity (%) = (A-B)/A × 100; where A and B are the rate of formazan formation in the absence and presence of sample, respectively.

**Assay of tissue nitric oxide (NO) concentration**

Direct quantitative measurement of NO level in the biological samples is very difficult, because it is a very labile molecule. In aqueous solution, NO reacts with molecular oxygen and accumulates in the plasma as nitrite (NO_2_^−^) and nitrate (NO_3_^−^) ions. Therefore, nitrite and nitrate, the stable oxidation end products of NO, can be readily measured in biological fluids and have been used *in vitro* and *in vivo* as indicators of tissue NO production [10]. In this study, the levels of NO metabolites were analyzed using a modification of the cadmium-reduction method [11]. This reaction using pre-treatment of samples reduces nitrate to nitrite, which can be accomplished by catalytic reactions using enzyme or cadmium. 500 μL of sample was deproteinized by adding 100 μL 30% ZnSO_4_ solution. Samples were stirred and centrifuged at 10,000 rpm for 10 min at 4 °C. Cd granules were activated using CuSO_4_ solution in glycine-NaOH buffer. After continuous stirring for 20 min, the samples were transferred to a microplate. The nitrite produced was determined by diazotization of sulfanilamide and coupling to naphthalene diamine. The absorbance was read at 540 nm using microplate reader. The nitrite concentration was determined with reference to a standard curve using 2.0 to 80 μmol concentrations of sodium nitrite, automatically. Data are reported as nmol NO/g wet tissue.

**Twenty-eight–day sub-chronic toxicity study**

**Body weight changes**

Average pre- and post-treatment body weights were measured, and change in weight was calculated at the end of the 28-day study for RNP^N^-treated, blank micelle-treated, TEMPOL-treated, and control groups.

**Assessment of blood pressure**

Blood pressure was measured pre- and post-treatment at 1, 7, 14, 21, and 28 days by the indirect tail-cuff method with a blood pressure monitor (model MK-1030, Muromachi Kikai, Tokyo, Japan).

**Assessment of hepatic function**

Blood samples were collected by intracardiac puncture on day 28 after behavior tests and blood pressure measurements. Then, plasma samples were obtained by centrifugation (6200 rpm, 2000 *g*, 10 min) of the blood. Plasma samples were assayed for aspartate aminotransferase (AST) and alanine transaminase (ALT) levels using a Fuji DRI-CHEM 3500 (Fuji-Film, Tokyo, Japan).

**Vital organ weight**

Qualitative data on weights of vital organs such as the liver, kidney, spleen, heart, lung, and testicle were assessed by carefully dissecting each organ from sacriﬁced animals into 10% neutral buffered formalin. Isolated organs were dried on paper towels and weighed on a sensitive weighing balance (XS105, METTLER TOLEDO, Greisensee, Switzerland). Each weighed organ was standardized from the body weight of each mouse.

**References**

1. Yoshitomi T, Hirayama A, Nagasaki Y. The ROS scavenging and renal protective effects of pH-responsive nitroxide radical-containing nanoparticles. Biomaterials. 2011;32(31):8021-8. Epub 2011/08/06. doi: 10.1016/j.biomaterials.2011.07.014. PubMed PMID: 21816462.

2. Yoshitomi T, Suzuki R, Mamiya T, Matsui H, Hirayama A, Nagasaki Y. pH-sensitive radical-containing-nanoparticle (RNP) for the L-band-EPR imaging of low pH circumstances. Bioconjug Chem. 2009;20(9):1792-8. Epub 2009/08/19. doi: 10.1021/bc900214f. PubMed PMID: 19685867.

3. Tang XN, Berman AE, Swanson RA, Yenari MA. Digitally quantifying cerebral hemorrhage using Photoshop and Image J. Journal of neuroscience methods. 2010;190(2):240-3. Epub 2010/05/11. doi: 10.1016/j.jneumeth.2010.05.004. PubMed PMID: 20452374; PubMed Central PMCID: PMC2898728.

4. McCord JM, Fridovich I. Superoxide dismutase. An enzymic function for erythrocuprein (hemocuprein). The Journal of biological chemistry. 1969;244(22):6049-55. Epub 1969/11/25. PubMed PMID: 5389100.

5. Goldblith SA, Proctor BE. Photometric determination of catalase activity. The Journal of biological chemistry. 1950;187(2):705-9. Epub 1950/12/01. PubMed PMID: 14803454.

6. Jakoby WB. Enzymatic basis of detoxication. New York: Academic Press; 1980.

7. Robertson RT, Hohmann CF, Bruce JL, Coyle JT. Neonatal enucleations reduce specific activity of acetylcholinesterase but not choline acetyltransferase in developing rat visual cortex. Brain Res. 1988;467(2):298-302. Epub 1988/04/01. PubMed PMID: 3378178.

8. Thompson RF. The brain : a neuroscience primer. 2nd ed. New York: W.H. Freeman & Co.; 1993. viii, 475 p. p.

9. Toda S, Kumura M, Ohnishi M. Effects of phenolcarboxylic acids on superoxide anion and lipid peroxidation induced by superoxide anion. Planta medica. 1991;57(1):8-10. Epub 1991/02/01. doi: 10.1055/s-2006-960005. PubMed PMID: 1648246.

10. Koltuksuz U, Irmak MK, Karaman A, Uz E, Var A, Ozyurt H, et al. Testicular nitric oxide levels after unilateral testicular torsion/detorsion in rats pretreated with caffeic acid phenethyl ester. Urological research. 2000;28(6):360-3. Epub 2001/02/28. PubMed PMID: 11221913.

11. Navarro-Gonzalvez JA, Garcia-Benayas C, Arenas J. Semiautomated measurement of nitrate in biological fluids. Clinical chemistry. 1998;44(3):679-81. Epub 1998/03/25. PubMed PMID: 9510886.

**Supporting Figures**

**
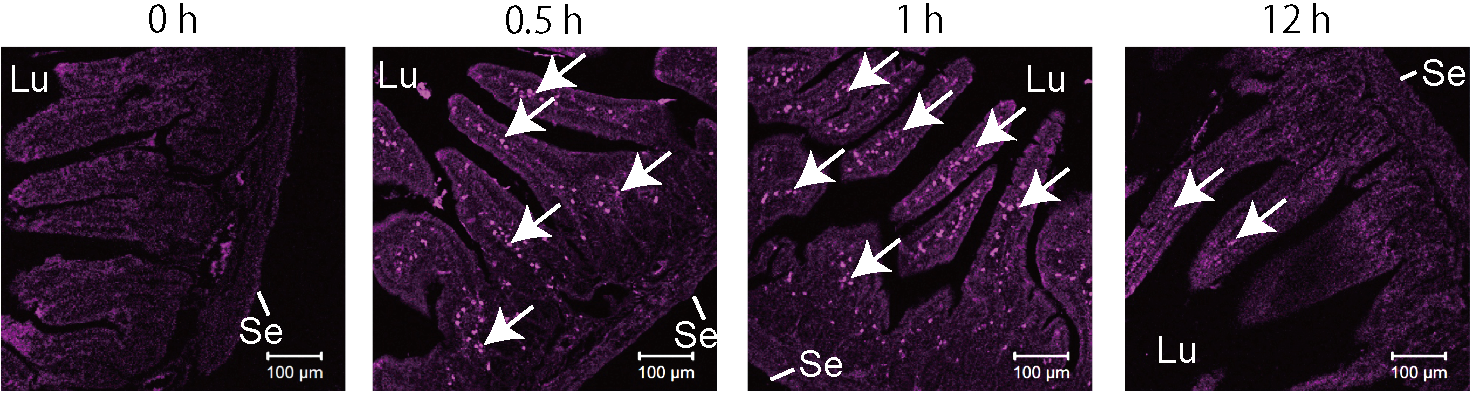
**

**Fig. A.** Time course of fluorescent image of the sections of duodenum after oral administration of Cy5.5-labeled RNP^N^. Mice were sacrificed at 0.5, 1, and 12 h after oral administration of 1 mL of Cy5.5-labeled RNP^N^ at a dose of 2 g/mL, and the duodenum section was cut circularly. The localization of Cy5.5-labeled redox polymer in the duodenum was analyzed by fluorescent confocal microscopy (Zeiss LSM 700 under oil immersion; Scale bars = 100 μm). Lu and Se in the figure indicate lumen and serosa, respectively. Arrows indicate fluorescent signal of Cy5.5-labeled redox polymer.

**Fig. B.** Photomicrographs of sections of (A) lung, (B) heart, (C) kidney cortex, (D) kidney medulla, (E) spleen, (F) liver and (G) testicle in SAMP8 mice histological stained with H&E at 10 × or 20 × magnification.

**Table A.** Effects of RNP^N^ on body weight change and vital organ weight of SAMP8 mice.

| Groups | Body weight increase change (g) | Lung (mg) | Heart (mg) | Liver (mg) | Kidney (mg) | Spleen (mg) | Testicle (mg) |
| --- | --- | --- | --- | --- | --- | --- | --- |
| Saline-treated SAMR1 mice | 0.539±0.040 | 10.594±1.671 | 5.822±0.452 | 66.651±3.998 | 19.677±0.932 | 3.274±0.340 | 5.423±0.169 |
| Saline-treated SAMP8 mice | 0.539±0.044 | 10.304±1.733 | 6.123±0.810 | 57.799±4.266 | 20.524±1.927 | 3.82±1.081 | 7.249±0.679 |
| Blank micelles-treated SAMP8 mice | 0.472±0.067 | 10.4±2.331 | 5.971±0.595 | 68.236±7.760 | 21.168±2.117 | 5.355±0.934 | 5.038±2.877 |
| RNP^N^-treated SAMP8 mice | 0.325±0.047 | 10.649±1.899 | 6.012±0.488 | 64.721±11.490 | 20.935±2.495 | 3.97±0.940 | 7.603±0.689 |
| TEMPOL-treated SAMP8 mice | 0.511±0.026 | 10.134±2.645 | 6.225±0.712 | 57.579±5.895 | 20.332±1.435 | 4.251±0.470 | 6.901±0.422 |

Values are expressed as mean ± SEM (n = 10).
